# Development of an information leaflet and diagnostic flow chart to improve the management of urinary tract infections in older adults: a qualitative study using the Theoretical Domains Framework

**DOI:** 10.3399/bjgpopen20X101044

**Published:** 2020-06-24

**Authors:** Leah Ffion Jones, Emily Cooper, Amelia Joseph, Rosalie Allison, Natalie Gold, Ian Donald, Cliodna McNulty

**Affiliations:** 1 Public Health England, Primary Care Interventions Unit, Gloucester, UK; 2 Nottingham University Hospital, Nottingham, UK; 3 Behavioural Insights, Public Health England, London, UK; 4 Gloucestershire Hospitals NHS Foundation Trust, Gloucester Royal Hospital, Gloucester, UK

**Keywords:** care home, diagnosis, anti-bacterial agents, self-care, prevention

## Abstract

**Background:**

Urinary tract infections (UTIs), older age, lack of access to health care, and recent antibiotic use are risk factors for *Escherichia coli* (*E. coli*) bloodstream infections.

**Aim:**

To explore the diagnosis and management of UTIs in primary care to inform the development of an information leaflet, a diagnostic flow chart, and recommendations for other resources.

**Design & setting:**

The study had a qualitative design and was undertaken in primary care settings and care homes.

**Method:**

Interviews and focus groups were informed by the Theoretical Domains Framework (TDF) with 31 care home staff, three residents, six relatives, 57 GP staff, and 19 members of the public. An inductive thematic analysis was used and themes were placed in the Behaviour Change Wheel (BCW) to recommend interventions.

**Results:**

Care home staff were pivotal for identifying suspected UTI, alerted clinicians to symptoms that influenced prescribing decisions, and reported confusion or behavioural changes as the most common diagnostic sign. Care home staff lacked knowledge about asymptomatic bacteriuria (ASB) and sepsis, and incorrectly diagnosed UTI using urine dipsticks. GP staff used urine dipsticks to rule out UTI and reported that stopping dipsticks would require a culture change, clear protocols, and education about ASB. Many prescribers believed that stopping urine dipstick use should help to reduce antibiotic use.

**Conclusion:**

A consistent message about ASB and UTI diagnosis and management in older adults should be communicated across the care pathway. Resource development should increase capability, motivation, and opportunity to improve management of suspected UTIs. An educational leaflet for older adults and a diagnostic flow chart for clinicians have been developed, and recommendations for interventions are discussed.

## How this fits in

*E. coli* rates have been steadily increasing in recent years with the highest rates of UTIs occurring in older adult populations. This study explored the context for diagnosis, treatment, and management of UTIs in older adults in care settings and general practice. An educational patient information leaflet for older adults on the topic of UTIs and a clinician-facing diagnostic flow chart have been developed, as well as a set of recommendations for further intervention development in this context.

## Introduction

Infection surveillance indicates that between 2015 and 2017 the rate of *E. coli* bloodstream infections (BSIs) in the UK increased from 70.5 to 76.8 per 100 000 population.^1^ Hospital surveillance has shown half of *E. coli* BSIs are related to the urogenital tract, and about half occur in people aged >75 years.^2^ Antibiotic treatment was the most commonly reported healthcare exposure in one-third of patients in the 4 weeks prior to BSI,^2^ and antibiotic resistance is at its greatest 1 month following antibiotic treatment.^3^ UTIs in ageing populations and in care homes are a significant problem. In a prospective Norwegian care homes study, healthcare-associated infections (HCAIs) occurred in 0.5% of residents per day; 38% were UTIs, and 94% received antibiotics.^4^

In institutionalised older adults, ASB prevalence can range from 15%–50%.^5,6^ Research has demonstrated that treating ASB in institutionalised older adults with antibiotics is associated with increased reinfection rates and isolation of increasingly resistant organisms in the recurrent infection when compared with no antibiotics.^7^ A 2017 systematic review of 30 studies reports almost half of ASB cases are managed inappropriately.^8^

Interventions developed using behavioural science have been shown to optimise antibiotic prescribing for common respiratory infections,^9^ and increases the acceptability and feasibility for end users.^10^ In a sample of 1415 primary care clinicians, over half reported using infection-related leaflets during consultations.^11^

The aim of this study is to identify opportunities to improve stewardship for UTI in older adults in community settings, by exploring the attitudes and experiences of older adults living in care homes, their relatives, care home staff, and general practice staff, in the diagnosis, management, and treatment of UTIs.

This study will inform the development of an educational leaflet for older adults and a diagnostic flow chart, and provide recommendations for further intervention developments for primary care.

## Method

### Choice of behavioural theory

For this study, the TDF^12^ and the BCW^13^ were chosen as the framework for exploration of behaviours around UTI management. The 14 TDF behavioural domains are designed to encompass all behavioural determinants and, therefore, provides a comprehensive framework to guide exploration. Key TDF domains can be applied to the BCW to further identify intervention techniques and intervention recommendations.

### Setting for qualitative data collection

The settings for data collection were English GP practices and care homes within Gloucestershire and Nottingham City. This allowed representation from a range of urban and rural, socioeconomic and ethnic groups.

### Participant selection and recruitment

General practice staff were selected to explore the current practice of diagnosing and managing UTIs in older adults in care or the community. General practices with higher, middle, or lower rates of local laboratory urine specimen submission in both regions were defined by dividing the practices across each clinical commissioning group (CCG) into three groups based on their submission rates. Practices with low numbers of older patients were excluded; for example, university practices.

Care home staff were selected to understand how they currently identify and manage UTIs in older adults in care. Care homes with a Care Quality Commission (CQC) rating of ‘at risk’ were excluded to avoid distractions from improvements to care.^14^ Care homes nearest to each GP practice recruited were invited to participate. Care home residents were then recruited through the care home staff. Practices and care homes were contacted by letter and then telephone until one facility per strata in each region was recruited. It was not always possible to recruit care homes connected to recruited general practices.

### Participant recruitment

#### Care homes and general practice staff

Managers were sent invitation letters from Public Health England (PHE), containing information sheets and consent forms. General practice staff, care home staff, residents with previous UTI and ability to consent, and relatives were invited to participate in interviews or focus groups. Non-responders received a telephone follow-up from the research team.

#### General public

Members of the PHE People’s Panel (500 members of the general public with research interests) were contacted to take part in focus groups to discuss their experiences of UTIs and provide their opinions on the older adult UTI leaflet. They were asked to participate if in the previous 2 years they had a self-reported UTI or had cared for someone with a UTI.

#### Stakeholders with a specialist interest in UTIs

Stakeholders were recruited from local CCGs and invited through an online professional community forum of practice for UTI (todipornottodip.slack.com).

### Data collection

The question schedule was informed by the TDF^12^ to understand the influences on participant behaviour (for example, their knowledge, skills, optimism and so on). Each question corresponds to one of the 14 behavioural domains, and each domain is covered at least once in the schedules. The topic areas discussed included participants’ attitudes and experiences of suspected and proven UTIs, antimicrobial resistance and antibiotics, and their opinions of the older adult UTI leaflet and the diagnostic flow chart. The questioning schedule was adapted and piloted with each participant group (see Supplementary Box S1). No significant changes were made following the pilots and thus pilot results are included in the results. Staff participants were offered 20 GBP gift vouchers and certificates in recognition of their participation, and the People’s Panel members were given 95 GBP towards their travel and costs.

Five female members of the research team conducted the data collection. Of these, two members have extensive experience in conducting interviews and focus groups, and the remaining three members worked under their guidance having helped facilitate in early interviews and focus groups. Focus groups were conducted in care homes or general practices. Care home residents with the ability to consent were interviewed on a one-to-one basis at their care home in a confidential setting, with the option of having a staff member or relative present. Stakeholder interviews were conducted by telephone or face to face depending on participant preference. The People’s Panel focus groups were conducted face to face in private rooms. Interviews and focus groups lasted from 30 to 90 minutes, were audiorecorded, transcribed verbatim, and checked for accuracy by two members of the research team. Transcripts were not returned to participants for comment.

Based on suggestions from each focus group or interview, iterative modifications were made to the leaflet and the diagnostic flow. The modified resources were presented at later focus groups and stakeholder consultation meetings.

### Data analysis

Data were analysed inductively^15^ by two researchers using NVivo (version 10).^16^ Ten per cent of transcripts were double-coded by a second researcher. Codes were discussed and an agreed consensus was reached. The themes were then discussed by the research group and were placed within the TDF framework.

Quotations from the transcripts are used in the results table to illustrate each TDF domain. These results were examined within the context of the BCW^13^ to identify intervention functions and behaviour change techniques relevant for intervention recommendations.

### Stakeholder consultation workshops and teleconferences

Excluding the research team, 24 experts in antimicrobial stewardship, UTI, older adult care, behavioural science, implementation, as well as GPs and patient representatives were invited through known contacts and snowballing to four stakeholder consultation meetings over the course of the data collection period: July 2017 teleconference, September 2017 workshop, November 2017 teleconference, and January 2018 workshop. Each meeting aimed to consolidate recommendations from the interviews and focus groups, cross reference these with the latest evidence and current practice, and agree a consensus for the resource content.

The meetings were structured so each resource was discussed line by line in an open forum; where consensus was not reached the majority consensus was used. Agreed changes were made to the leaflet and diagnostic flow chart following each meeting, and were used in subsequent focus groups and interviews.

## Results

### Sample characteristics

**Figure 1.**
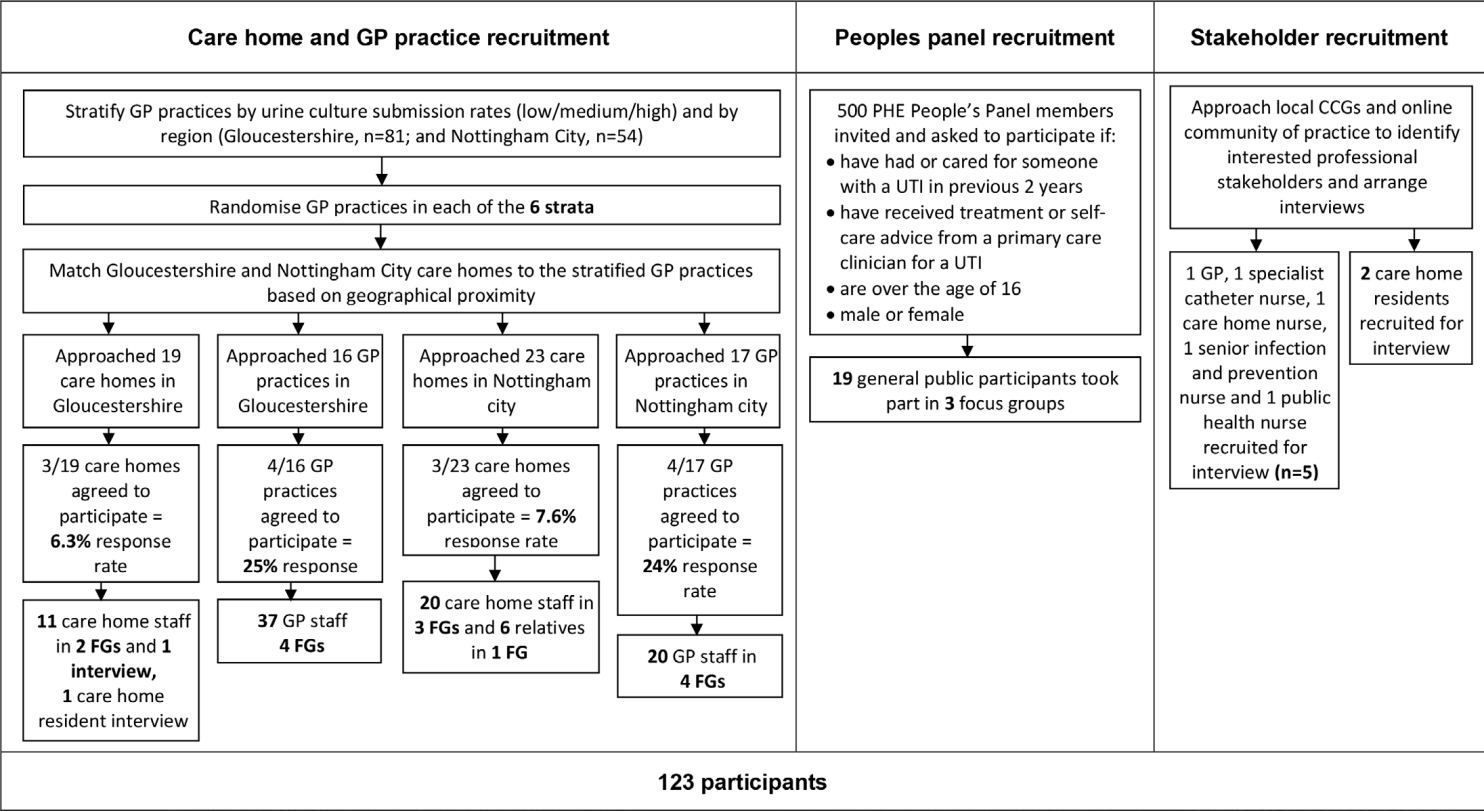
Recruitment flow chart. CCG = clinical commissioning group. FG = focus group. PHE = Public Health England. UTI = urinary tract infection.

In Gloucestershire, 25% (*n* = 4/16) of practices and 16% (*n* = 3/19) of care homes approached agreed to participate, and in Nottingham 24% (*n* = 4/17) of practices and 13% (*n* = 3/23) of care homes agreed to participate. There were eight focus groups, comprising 28 GPs, 17 nurses, and 12 other staff (20 from Nottingham and 37 from Gloucestershire), see Figure 1. The most common reason for non-participation was staff lack of time, and lack of capacity for care home residents.

Two participating care homes had <10 residents, and four had 11–49 residents. Thirty-one care home staff (one nurse, two care home managers, and 28 care assistants) took part in five focus groups and one interview (11 from Gloucestershire and 20 from Nottingham City). All participating care homes were privately owned, apart from one not-for-profit care facility in Nottingham. Six residents' relatives participated in one focus group in Nottingham City. Three residents were interviewed.

Nineteen PHE People’s Panel members aged 36–70 years took part in three focus groups. Five stakeholders were interviewed: one GP, one catheter nurse, one care home nurse, one senior infection and prevention nurse, and one public health nurse. Additionally, six members of the research team and 24 individuals from professional and patient interest groups participated in four stakeholder meetings.

Data analysis identified domains from the TDF (see Supplementary Table S1) that influence behaviour in care home and general practice settings around the diagnosis and management of UTIs.

### Diagnosing suspected UTIs

#### Identification and awareness of symptoms

Care homestaff displayed basic UTI knowledge, such as knowing a UTI is an infection in the urine or 'water system'. Knowing a resident well and being familiar with their usual behaviour and body language was described by most care home staff groups as being important to recognise early indications of infection. Most suggested that they would rely on symptoms to diagnose a UTI, and explained that; in most care homes, it is the care home staff that identified initial signs and symptoms and then notified the nursing team for further action to be taken, if necessary. All care home staff identified confusion and behavioural changes as the most common symptoms, especially for residents with dementia. All care home staff groups were able to identify some other symptoms indicative of a potential UTI: smelly and discoloured urine, pain, temperature, lethargy, irritability, incontinence, confusion, discomfort, loss of appetite and/or thirst, changes in body language and/or changes in facial expressions, changes in continence frequency, hallucinations, not passing urine, pale skin, and wobbly on their feet and/or unable to stand. Only one individual suggested residents with a UTI can be asymptomatic.

Members of the public and relatives of older adults identified UTI symptoms in older adults, such as: new confusion, not feeling well, wetting themselves, burning sensation, temperature, pressure in lower back, regular toileting, difficulty passing urine, delirium, staggering, and weakness.

#### The challenge of collecting urine samples

When care home staff suspected a UTI, attempts were usually made to collect a urine sample, which could be difficult especially in residents with incontinence or dementia. Staff described using a commode, a disposable kidney bowl or obtaining a sample from a used incontinence pad. Most general practice staff suggested patients who were catheterised or incontinent were a challenge as care home staff were not always trained to obtain appropriate urine samples. A catheter nurse reported lack of guidelines for specimen collection in care homes.

#### Urine dipsticks as a diagnostic tool

All care homes reported using urine dipsticks to confirm their suspicions of a UTI in their residents (some at the request of GP practices), either in the care home itself, or by providing urine samples to the GP practice for testing. Only some care home staff knew dipstick results were not conclusive of UTI without the addition of localising symptoms.^17^ Only one care home staff member shared that they were aware of ASB and that urine dipsticks cannot distinguish between ASB and clinical UTI. Staff from one care home described placing dipsticks directly onto incontinence pads to test the urine.

#### Barriers in diagnosing UTIs in older adults

All general practice staff discussed the difficulty in identifying UTIs in older adults, especially when many older adults have only some of the non-specific diagnostic symptoms, such as incontinence or confusion. Most general practice staff felt that care home staff do not want to be held accountable for missing a serious infection and, therefore, expect antibiotics to be prescribed. GP staff had mixed views on the value of using urine dipsticks to diagnose UTIs in older adults. Some relied on dipsticks to confirm the diagnosis. GP staff reported that they were unreliable in older adults owing to the high ASB prevalence, but some stated they would rather have a test than no test, and a negative result could ‘rule-out’ UTI. Some GP staff agreed that stopping the urine dipstick use could increase appropriate treatment and save money. However, others were concerned that changing the culture of using urine dipsticks in care homes would be difficult. Some feared that without urine dipsticks, GPs would have to visit care homes more regularly to assess patients.

Many care home staff found it difficult to identify the cause of confusion and/or delirium or type of infection suspected, which led to increased reliance on urine dipsticks and a defensive management style. Some care home staff suggested their lack of medical training was a barrier here and they were interested in learning about the acronym PINCH ME (Pain, Infection, Nutrition, Constipation, Hydration, Medication, Environment) used in the differential diagnosis of delirium.^18^

#### Sepsis

Few discussed considering pyelonephritis or sepsis, but all referred residents to nursing or GP staff if they were concerned. Some care home staff were unable to identify sepsis symptoms and were surprised that having trouble breathing and cold skin are signs.

#### Urine culture

Practices and care homes had varied policies and processes in place for submitting a urine sample for culture in suspected UTI. This was influenced by GP requests, lab urine specimen collections and turnaround times, ease of obtaining lab results, and lack of influence on patient outcomes. When GP staff were informed that antibiotic resistance and *E.coli* BSIs had increased, some GP staff agreed it would be useful to send a urine for culture in all patients aged >65 years when prescribing an antibiotic.

### Managing and caring for UTIs: antibiotics, medication, and alternatives

Some care home staff expected that an antibiotic would always be prescribed for a suspected UTI. Staff from one care home believed their residents were less likely to have resistant UTIs because they were a residential home, rather than a nursing home. Some relatives reported that antibiotics did not work for their relative receiving care.

Some care homes reported providing paracetamol to residents with UTI, especially if they had a high temperature. Two residents said they were not given any self-care advice from their GP when diagnosed with a UTI.

Some relatives reported that their older adult relatives were reluctant to admit they needed help, and they were more inclined to take advice from health professionals, rather than friends and family. Effective methods of self-care treatments reported by care home staff, residents, and relatives included cranberry juice and lemon barley water.

### Preventing UTIs

#### Awareness of prevention strategies

Many care home staff mentioned the importance of maintaining good personal hygiene to prevent future UTIs; for example, cleaning after toileting, wiping from front to back, and changing incontinence pads regularly. Many of the staff referred to cranberry juice as a way of managing or preventing a UTI. One member of care home staff reported not having enough information available around prevention of UTIs and many participants requested that any patient-facing information should include prevention.

#### The importance of hydration

All care home staff discussed the importance of hydration in managing and preventing UTIs, all agreed that providing residents with their preferred drink facilitated hydration, and all care homes mentioned providing a variety of drink options. However, most care home staff expressed difficulty in getting residents to drink enough. Several care homes tried to encourage hydration further by providing water-based foods for residents such as sliced fruit, jellies, and soups. All residents were mindful of staying hydrated; although, one did not know why this was important. One care home mentioned that they encourage relatives to promote hydration with residents, and relatives mentioned that they encourage drinking when they visit. Some general practice staff and members of the public reported dehydration in older adults was common. They reported that this was owing to older adults deliberately not drinking because they have difficulty going to the toilet; this was also reported by care home staff. All participants reported that a section on hydration in any patient-facing information would be useful.

### Leaflet and flow chart modifications

#### Leaflet modifications

The leaflet has been made sex neutral; a sex-neutral illustration of the urinary system has been included on the front page of the leaflet as the diagram was seen to be useful for illustrating locations of infections, and helps older adults understand their condition’s physiology. A section covering other causes of confusion has been included, as relatives particularly valued understanding other potential causes. The signs and symptoms have been divided into sections for those with and without catheters as care home residents with catheters felt some of the symptoms were not relevant for them.

The importance of hydration was a key finding and has, therefore, been emphasised in the leaflet. Lack of knowledge around effective prevention strategies has been addressed by including statements of strategies where evidence is sparse; for example, cranberry products. The antibiotic-resistance section has been simplified and made suitable for older adults with difficulty understanding the concept.

All participants reported that increasing the font size for those with poor eyesight, information chunking into coloured sections, and use of pictures would facilitate reading and understanding.

#### Flow chart modifications

Information on ASB and the unreliability of urine dipsticks has been highlighted in the diagnostic flow chart to ensure consistency with the leaflet and the National Institute for Health and Care Excellence (NICE).^19^ The PINCH ME^18^ acronym and information on other infections have been included in the flow chart to highlight other things that cause delirium and/or confusion in older adults. References for taking urine samples to prevent contamination have also been included.

## Discussion

### Summary

Despite the majority having no formal medical training, care home staff play an important role in the early identification of infection in care home residents. Their understanding and subsequent communication of signs and symptoms to nurses or GPs are likely to influence whether antibiotics are prescribed and the early diagnosis of pyelonephritis or sepsis. However, care home staff have a limited knowledge of UTIs, sepsis, and ASB, and therefore lack understanding of the limitations of using urine dipsticks to aid diagnosis. Common barriers to best practice faced by care home staff include difficulties in obtaining urine samples from some residents, having no guidelines for taking samples from catheters, having to chase urinalysis results, lack of information around prevention, and feeling pressured to dipstick by GPs. This study found that to increase staff capability, such as their knowledge and skills, a clear consistent message about UTI diagnosis and management in older adults needs to be communicated appropriately across the care pathway, including clear guidance for care homes, and general practice to not use urine dipsticks as a diagnostic tool for suspected UTI in older adults.

Findings from the BCW suggest a multi-faceted intervention could include the provision of diagnostic guidelines, including advice on obtaining urine samples, and educational resources to increase capability and motivation. This may help to overcome the culture change required and reinforce the use of these guidelines. The guidelines should include role-appropriate content to increase gaps in knowledge and skills around the diagnosis of suspected UTI; for example, ASB and lack of value of urine dipsticks; sepsis; the differential diagnoses including causes of delirium; appropriate urine culture self-care; safety-netting; and prevention of UTIs identified in this study. These resources should align with patient-facing resources on UTIs for older adults and their relatives, such as leaflets and posters.

Based on the findings, the leaflet and flow chart have been modified in line with the suggestions reported in the results section.

### Strengths and limitations

There were difficulties in recruiting care home residents to the study owing to lack of capacity in most residents; therefore, two residents were interviewed from another CCG region, and treatment was discussed with older members of the general public in the PHE focus groups. Many care homes and GP practices declined to participate; therefore, a biased sample with more knowledge may have been interviewed. Indeed, care home staff were not spoken to from NHS or local authority owned facilities (these only account for 2% of the facilities); however, there was a broad range of knowledge and skills across practices, data saturation was reached, and the key findings were generally consistent across care homes and practices.

The older adult leaflet and the diagnostic flow chart were developed using a novel cycle of iterative modifications following interviews, focus groups, and stakeholder consultation meetings, to ensure scientific and clinical accuracy in the content, and to ensure suitability for the target audience.

### Comparison with existing literature

A qualitative study of physicians and nurses working in long-term care facilities found nurses play an integral role in the identification of symptoms and subsequent prescribing behaviours of clinicians. The authors suggest that interventions to reduce antibiotic prescribing in this group need to target nursing staff directly involved in residents’ care.^20^ Additionally, they describe a culture of prescribing antibiotics for ASB and suggest further education is needed. Antibiotic prescribing for ASB was also corroborated in an Irish study using the TDF; the authors suggested auditing and benchmarking antibiotic usage against other care homes, guideline provision, education, and training.^21^ However, both studies did not interview carers who also play an important role in reducing treatment for ASB.^20,21^ A review by Nicolle supports these conclusions but argues education alone will not be enough to improve antibiotic use in this group;^22^ more targeted diagnostic testing to distinguish between a clinical UTI and ASB is needed.

### Implications for research and practice

The key findings identified correspond to the TDF domains of lack of knowledge and skills, professional role of care home staff in UTI identification, the social influence between carers and general practice staff, and the environmental context and lack of resources. Table 1 shows how these TDF domains link to the BCW,^13^ allowing for identification of intervention functions and behaviour change techniques to inform intervention recommendations in this context. Finding 7 in Table 1 aligns with the recommendation to develop an information leaflet, which has been a key study outcome.

**Table 1.** Intervention recommendations using the Theoretical Domains Framework, the Behaviour Change Wheel, and Behaviour Change Techniques.

Finding	TDF domains	COM-B (Capability, Opportunity, Motivation - Behaviour)	Intervention functions(selected)	Behaviour Change Techniques (selected)	Recommendations and examples
1. Care home staff lack knowledge of UTI and ASB	Knowledge	Psychological capability	Education	Information about social and environmental consequences;Information about health consequences;Feedback on outcomes of the behaviour;Information about antecedents;Credible source;Adding objects to the environment	Education on: asymptomatic bacteriuria and evidence of diagnosis of UTIs in >65 seconds; the harm of inappropriate antibiotics; and risks of antimicrobial resistance in care home residents. Diagnostic tools, such as flow charts, designed for use by carers to help accurately identify UTIs without the use of dipsticks.
2. Lack of awareness for care home staff of limitations of urine dipsticks in the diagnosis of UTIs in older people	Knowledge	Psychological capability	Education		
3. Care home staff identify initial signs of infection	Professional role and identity	Reflexive motivation	Education;Persuasion		
4. Care home staff feel pressure from GPs to use dipsticks and GPs believe it will be difficult to change the culture of dipsticking	Social influence	Social opportunity	Environmental restructuring		
5. Difficult to obtain urine samples from some residents	Skills	Physical capability	Training	Instructions on how to perform a behaviour;Demonstration of the behaviour;Problem solving;Action planning	Clear and comprehensive guidance on taking urine samples in >65 seconds, including apparatus required. Education and training on the guidance, including taking urine samples to prevent contamination.
6. There are no guidelines for taking urine samples from catheters in care homes	Environmental context and resources	Physical opportunity	Training;Enablement		
7. Not enough available information for carers on prevention of UTI	Environmental context and resources	Physical opportunity	Environmental restructuring;Enablement	Adding objects to the environment	Resources to educate care home staff and residents on prevention of UTI; for example, leaflet.
8. Care homes have to chase urinalysis results	Environmental context and resources	Physical opportunity	Environmental restructuring;Enablement	Restructuring the physical environment;Social support (practical)	Streamlined process of sending urine samples to the lab; for example, care home collection or drop-off.

ASB = asymptomatic bacteriuria. TDF = Theorectial Domains Framework. UTI = urinary tract infection.

Care home staff need to access clear guidelines for obtaining urine samples in patients who are catheterised. There are existing national guidelines,^23^ with practical step-by-step guidance^24^ that can be signposted to or used to develop new resources. Local protocols should advise that urines are only sent if antibiotics are prescribed in symptomatic cases, with the appropriate clinical details provided, to prevent overtesting and overdiagnosis. National guidance recommends urine culture in all patients aged >65 years who are prescribed antibiotics,^25^ but clinicians should not delay empirical antibiotics in symptomatic patients.^25^ The process for receiving the results needs to be timely in order for carers and clinicians to modify treatment if needed.

The To Dip Or Not To Dip quality improvement programme is designed for use in care homes and should lead to improved diagnoses and communication.^26^ Dipstick use, antibiotics, leaflets, cultures, and *E. coli* BSIs could be audited in the practice to assess guideline use,^27^ detect any unexpected consequences, and motivate staff.

A diagnostic flow chart and a patient-facing leaflet for patients aged >65 years with consistent messaging have been developed based on findings from this study, and are now published by PHE, the authors, and the Royal College of General Practitioners (RCGP), for national and international use on the RCGP website (www.rcgp.org.uk/targetantibiotics).^25,28^

Barriers to resource implementation are staff lack of time and social pressure to provide dipstick results. Staff will need to be motivated to use the resources through education and routine feedback about antibiotic use, UTI, and *E. coli* BSI rates. Tools that provide a consistent approach to improve communication between the care home and GP practice will, therefore, be needed.

A full mixed-methods evaluation of the UTI leaflet for older adults,^29^ the UTI diagnostic tools,^30^ and other PHE UTI resources is needed to assess their impact on UTIs, antibiotic use, and hospital admissions.
